# The Status and Future Directions of Treatments for Polyglutamine Spinocerebellar Ataxia: A Bibliometric and Visual Analysis

**DOI:** 10.2174/011570159X360111250502055242

**Published:** 2025-05-19

**Authors:** Siyu Ding, Linliu Peng, Zhao Chen, Mengyuan Dong, Cuiling Tang, Yiqing Gong, Lang He, Qi Wu, Rong Qiu, Hong Jiang

**Affiliations:** 1 Department of Neurology, Xiangya Hospital, Central South University, Changsha, Hunan, 410008, China;; 2 Bioinformatics Center and National Clinical Research Center for Geriatric Disorders, Xiangya Hospital, Central South University, Changsha, Hunan, 410008, China;; 3 Hunan International Scientific and Technological Cooperation Base of Neurodegenerative and Neurogenetic Diseases, Changsha, China;; 4 School of Computer Science and Engineering, Central South University, Changsha, China;; 5 Department of Neurology, The Third Xiangya Hospital of Central South University, Changsha, Hunan, 410008, China;; 6 Key Laboratory of Hunan Province in Neurodegenerative Disorders, Central South University, Changsha, Hunan, 410008, China;; 7 National International Collaborative Research Center for Medical Metabolomics, Central South University, Changsha, Hunan, China;; 8 Furong Laboratory, Central South University, Changsha, Hunan, 410008, China;; 9 Brain Research Center, Central South University, Changsha, Hunan, 410008, China

**Keywords:** Bibliometrics, polyglutamate spinocerebellar ataxia, treatment, VOSviewer, CiteSpace, gene therapy

## Abstract

**Background:**

Polyglutamine (polyQ) spinocerebellar ataxias (SCA) are a group of autosomal dominant neurodegenerative disorders for which no effective treatments currently exist. These conditions impose a significant burden on patients, their families, and society. Consequently, the treatment of these disorders has attracted significant global interest.

**Objective:**

We conducted this bibliometric analysis to identify the key research hotspots and predict the future research directions of this field.

**Methods:**

Studies relating to the treatment of polyQ SCA published from 1999 to 2024 were retrieved from the Web of Science Core Collection database. Relevant papers were selected using predefined inclusion and exclusion criteria. HistCite, VOSviewer, CiteSpace, and alluvial generator were used in the bibliometric analysis.

**Results:**

Overall, 935 papers were included. The number of publications in this field showed a trend toward a fluctuating increase. The United States and the University of Coimbra were the leading countries and institutions, respectively, in terms of publication number. The two most productive and highly cited authors were Luis Pereira de Almeida and Patricia Maciel. The journals *Cerebellum*, *Human Molecular Genetics*, and *Movement Disorders* were considered the most influential based on the number of publications and citations. Furthermore, “new SCA types”, “Huntington’s disease”, “clinical trial”, “gene therapy”, “disease models,” and “Aggregation clearance therapy” emerged as current hotspots in this field, as revealed by the reference and keyword analyses.

**Conclusion:**

This study presents a systematic bibliometric analysis of research on the polyQ SCA treatment, which we hope will assist researchers in identifying the key topics and future research directions in this field.

## 1 INTRODUCTION

Polyglutamine (polyQ) spinocerebellar ataxia (SCA) constitutes a large group of progressive autosomal-dominant neurodegenerative disorders [1]. The most prevalent types of polyQ SCA, which include SCA1, SCA2, SCA3, SCA6, SCA7, SCA17, and dentatorubral pallidoluysian atrophy (DRPLA), share a common pathological mechanism characterized by the expansion of a glutamine-encoding CAG repeat within the affected gene [1-3]. Some newly discovered subtypes of SCA, such as SCA51/THAP11, are also classified as polyQ SCA [4]. The prevalence of the various types of polyQ SCA varies across different regions and ethnicities, but it typically ranges from 1 to 3 cases per 100,000 individuals [5]. In patients with polyQ SCA, disease severity progressively worsens, leading to a shortened lifespan compared to the general population [1]. Although the incidence rate of polyQ SCA is relatively low, the total number of patients with this condition is significant owing to the large population base and the high genetic heterogeneity of polyQ SCA. These disorders, therefore, pose a significant burden to both patients and their families and, more broadly, to society.

Although recent insights have deepened our understanding of the mechanisms underlying polyQ SCA, there are still no effective treatments for this group of disorders [2, 3, 6]. There are two primary strategies for treatment: reducing the levels of toxic gene products and using disease-modifying therapies [1]. Some agents have shown therapeutic potential, such as antisense oligonucleotides (ASOs) [7-12], small interfering RNAs (siRNAs) [13, 14], artificial microRNAs (miRNAs) [15, 16], and CRISPR-Cas nucleases [17-19] which have been shown to reduce pathogenic gene products. Additionally, pharmaceutical drugs, such as riluzole [20-23], valproic acid [24-26], varenicline [27, 28], and lithium carbonate [29-31] have shown promise in both clinical and preclinical studies. Supportive treatments, including coordinative physical therapy [32-35] and transcranial magnetic stimulation [36-39], have also demonstrated their effectiveness in alleviating the symptoms of ataxia across multiple studies. Despite significant advancements made for the treatment of polyQ SCA, challenges remain, such as how best to negate the harmful off-target effects of gene therapies [40, 41], determining the optimal timing of treatment, and maintaining treatment effects in the long term [42]. Consequently, additional research is essential to discover new therapeutic methods and improve the effectiveness of existing ones.

Bibliometric analysis is a quantitative methodology that offers statistical insights into research outputs within a specific field, thereby aiding in the assessment of the impact of studies and forecasting future research trends [43]. Compared to conventional qualitative analysis, bibliometric analysis enhances the efficiency of knowledge acquisition, provides insights into the academic quality and influence of journals and authors, and measures the strength and impact of countries and institutions in a given research field. This approach has been effectively applied in various fields, such as diabetes mellitus [44-46], cancer [47, 48], and neurodegenerative diseases [49-51] research. Additionally, bibliometric analysis has been used to evaluate the status and future directions in the fields of rare hereditary diseases, such as hepatolenticular degeneration [52] and hereditary hearing loss [53]. However, bibliometric analysis has not yet been utilized for the quantitative evaluation of research on polyQ SCA treatment.

In this study, we conducted a bibliometric analysis of research on polyQ SCA treatment using tools such as VOSviewer (Leiden University, the Netherlands), CiteSpace [54], R, and alluvial generator (https://www.mapequation.org/apps/AlluvialGenerator.html). Through this analysis, we evaluated the distribution of related publications by country, institution, author, and journal. Additionally, we assessed the keywords of these publications to identify research hotspots and forecast future trends in this field. Through this study, we aim to provide researchers with a comprehensive understanding of the current research landscape and emerging areas of interest within the domain of polyQ SCA treatment.

## 2 MATERIALS AND METHODS

### 2.1 Data Sources and Search Strategies

Relevant publications were sourced from the Web of Science Core Collection (WOSCC) database, which is widely recognized as the most used resource for bibliometric analysis. Papers published up to December 2, 2024, were retrieved from the WOSCC. The search strategy is detailed in Fig. (**1**) and Table **1**. We searched for papers reporting treatments for all known polyQ SCA subtypes, including SCA1, SCA2, SCA3, SCA6, SCA7, SCA17 and DRPLA. In addition, we conducted separate searches for treatments specific to each of these SCA subtypes. Furthermore, we searched for research on treatments for the newly identified SCA51/THAP11. However, no studies meeting the inclusion criteria were found. To broaden the search scope, keywords, and supplementary terms were derived from the MeSH database on PubMed. Only English-language publications were included in the analysis.

### 2.2 Data Screening

Two researchers independently reviewed and assessed the relevant publications in sequence, with any disagreements resolved by a third researcher through reassessment. The inclusion criteria for the publications were: (1) original articles or reviews; (2) a primary focus on the treatment of polyQ SCA, including clinical or experimental research; and (3) English-language publications. The exclusion criteria were: (1) topics not related to the treatment of polyQ SCA; (2) literature types other than original articles or reviews, such as letters or books; and (3) non-English publications. The author, title, source, and abstract of each record were downloaded as .txt files from the WOSCC database for subsequent analysis. The search results are presented in Table **1**.

### 2.3 Statistical Analysis and Visualization

Bibliometric analysis was performed using several tools, including Microsoft Office Excel 2016, HistCite (version 12.3.17), VOSviewer (version 1.6.19), CiteSpace (version 6.1.6), the R package Bibliometrix (version 4.1.3), and the alluvial generator. As well as dataset analysis, these tools were used for visual analysis, and the results are presented as graphs, networks, charts, and maps.

The number of productions is depicted using tables and bar graphs, which were generated using Microsoft Excel 2016.

HistCite [55] is a software tool that was designed to analyze and visualize direct citation linkages among scientific papers, producing clear and informative tables and graphs from Web of Science data. This capability enables researchers to track the progressive development of a field and to identify key articles and contributors. In this study, both the Total Local Citation Score (TLCS) and the Total Global Citation Score (TGCS) were extracted using HistCite. TLCS quantifies the total citations that a publication has received within the dataset, indicating its significance in the respective field. The TGCS quantifies the total citations across all records in the Web of Science, informing of the most influential research globally.

VOSviewer [56], a software tool designed to analyze bibliometric data and construct bibliometric networks, was utilized to analyze bibliographic data by country, author, institution, and journal. A co-citation analysis involving the cited authors and journals was performed. The results are displayed as visual networks where each item is denoted by a circular label. The size of each circle is determined by the item’s weight, with larger circles indicating heavier weights. The items in the network are grouped into clusters and differentiated using various colors. The connecting lines between the circles represent the links among the items.

CiteSpace [54] is a software tool designed to analyze trends and patterns in research across various fields. CiteSpace was used to construct cluster networks and timeline graphs, as well as to analyze bursts in references and keywords. Within the network, the size of each dot reflects its significance or prominence, with larger nodes indicating greater influence. The colors of the nodes correspond to the tree-ring history pattern, where each color denotes the publication timeline or the emergence of a concept, with those receiving significant attention highlighted in red. Node connectivity was quantified using betweenness centrality, with higher values indicating more connections. Nodes with a betweenness centrality exceeding 0.1 are marked with a purple ring. Additionally, the structure of the network was assessed using Modularity Q and silhouette values, with higher modularity values indicating well-defined clusters. Clusters with a Modularity Q greater than 0.3 and a mean silhouette value above 0.5 were considered robust and coherent.

Bibliometrix [57] is an R package that offers a comprehensive range of bibliometric analysis methods. Bibliometrix was used to compute the h-index and g-index for the authors and publishing journals. The h-index measures both the quantity and influence of citations received by a scholar or journal, with a higher h-index indicating a greater academic impact. However, since the h-index may not fully reflect the influence of highly cited papers, the g-index was also calculated to provide a more complete assessment of citation impact.

The alluvial generator (https://www.mapequation.org/apps/AlluvialGenerator.html) was used to create alluvial plots. A co-occurrence network of keywords extracted from CiteSpace was input into the alluvial generator to produce these maps. Within the alluvial map, each keyword is treated as a node, and each column symbolizes either a time slice or a specific type of SCA. The nodes within each column are grouped into clusters, with each cluster treated as a module. The nodes in adjacent columns are interconnected with lines, with the longest connected nodes identified as significant and highlighted in varying colors for emphasis.

## 3 RESULTS

### 3.1 Performance Analysis of Publications on polyQ SCA Treatment

#### 3.1.1 General Analysis of Publications on SCA Treatment

##### 3.1.1.1 Distribution of Publications by Year

**Fig. (2):**
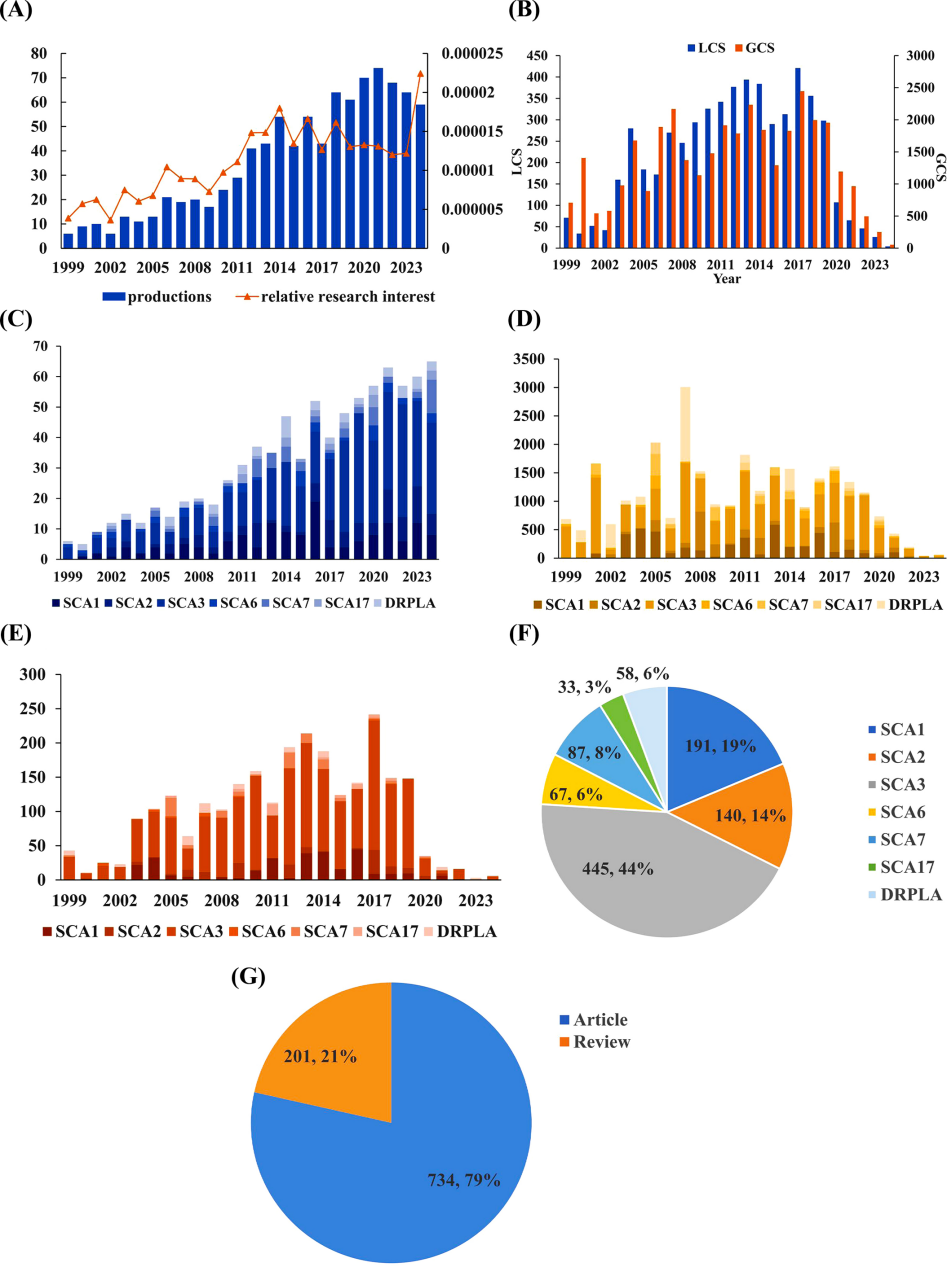
The global trends of and categories of studies in the field of treatment to polyQ SCA (**A**) The annual number and relative research interest of productions related to treatment to polyQ SCA from 1999 to 2024. (**B**) The annual GCS and LCS of productions related to treatment to polyQ SCA from 1999 to 2024. (**C**) The annual number of productions related to treatment to different types of polyQ SCA from 1999 to 2024. (**D**) The annual GCS of productions related to treatment to every type of polyQ SCA from 1999 to 2024. (**E**) The annual LCS of productions related to treatment to every type of polyQ SCA from 1999 to 2024. (**F**) The total number of productions related to treatment to every type of polyQ SCA. (**G**) The total number of productions related to treatment to polyQ SCA in different types of literature.

Overall, 1420 papers were sourced from the WOSCC database. The data screening strategy is depicted in Fig. (**1**). Of the 1,420 identified papers (Fig. **2**), 935 papers were included for further analysis, including 734 articles and 201 reviews (Fig. **2G**) published between 1999 and 2024. Fig. (**2A**) shows that the publication trend for studies reporting treatments for polyQ SCA varied, showing fluctuating growth from 1999 to 2021 and stabilizing at approximately 64 publications per year from 2022 to 2024. Additionally, the relative research interest (RRI), defined as the proportion of papers in this field relative to those in all fields annually, also fluctuated, reaching its highest point in 2024.

To assess the impact of the papers included in this study, the GCS and LCS were analyzed. Fig. (**2B**) illustrates that the annual GCS fluctuated at around 1482 from 1999 to 2017, and it subsequently decreased in more recent years. The GCS peaked in 2017 with a total of 2446 citations. Similarly, the LCS generally increased from 1999 to 2017, followed by a decline to 2024. The highest LCS was recorded in 2017, with 421 citations in total.

##### 3.1.1.2 Distribution of Publications by SCA Subtype

The number of publications pertaining to different subtypes of SCA was analyzed in Fig. (**2F**). Studies on the treatment of SCA3 accounted for 44% of the total publications, making it the most frequently studied type of SCA, followed by SCA1 and SCA2. SCA3 was first studied in 1999, making it the earliest researched SCA subtype. Conversely, studies on other subtypes of polyQ SCA, specifically SCA6, SCA7, and SCA17, were comparatively fewer in number. The number of annual publications on SCA1, SCA2, and SCA3 demonstrated fluctuating growth, with SCA3 experiencing the most significant increase (Fig **2C**). Research on other subtypes of polyQ SCA showed sporadic publication activity.

The GCS and LCS for the various types of SCA are displayed in Figs. (**2D**, **2E**), respectively. The LCS and GCS for each type of SCA varied annually. Notably, studies on SCA3 demonstrated the highest LCS and GCS among all studies. Specifically, the LCS for SCA3 peaked in 2019 with 172 citations, while its GCS peaked earlier in 2007 with 1,437 citations. Furthermore, studies on SCA1 and SCA2 ranked second and third, respectively, for both the LCS and the GCS, similar to their publication frequencies.

#### 3.1.2 Analysis by Country

From 1999 to 2024, researchers from 28 countries investigated treatments for polyQ SCA. According to Fig. (**3A**) and Table **2**, the United States, China, Japan, Portugal, Germany, England, the Netherlands, France, India, and Brazil were identified as the top 10 countries with the highest research output. The geographical distribution of these publications is illustrated on the World Map in Figs. (**3C**, **3D**) displays the annual number of publications from the five most productive countries. Analysis of these data revealed that the research output of these leading countries exhibited fluctuating growth, with a peak in 2018 at 49 publications.

**Fig. (3):**
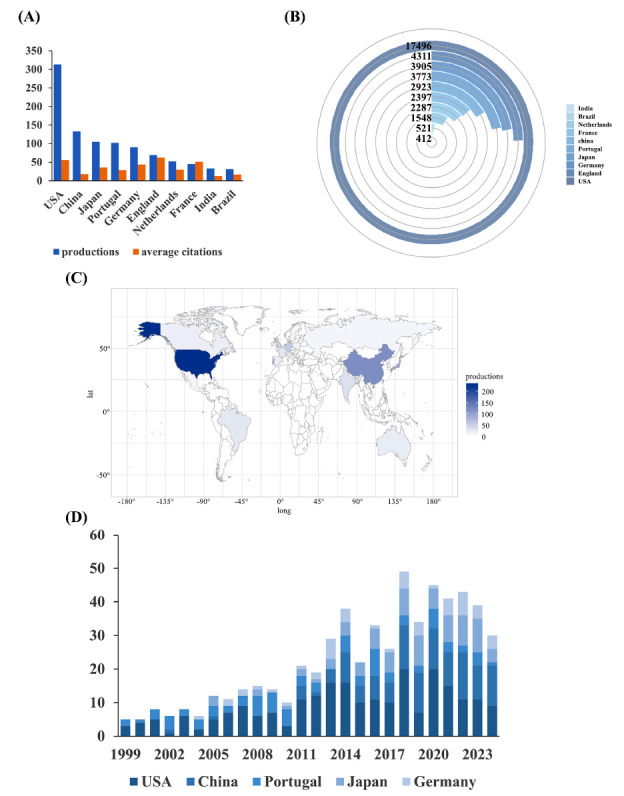
The distribution of studies in different countries (**A**) The total number and average citation number of studies related to treatment to polyQ SCA in top 10 most productive countries. (**B**) The total total citation number of studies related to treatment to polyQ SCA in top 10 most productive countries. (**C**) A world map depicting the distribution of studies related to treatment to polyQ SCA. (**D**) The annual productions of studies related to treatment to polyQ SCA in top 5 most productive countries from 1999 to 2024.

We analyzed the citation counts of the top 10 productive countries/regions (Fig. **3B**). The United States was the leading contributor, with a total of 17,496 citations, averaging 55.90 citations per paper. Following closely, England ranked second, accumulating 4,311 citations, averaging 62.48 citations per paper. Germany ranked third with 3,905 total citations, averaging 43.39 citations per paper.

#### 3.1.3 Analysis by Institution

Using VOSviewer, we observed that a total of 116 institutions participated in studies on the treatment of polyQ SCAs. The production and number of citations of the top 25 institutions are detailed in Figs. (**4A** and **4B**) and in Table **3**. The University of Coimbra was the most productive institution, with 50 productions and 1,767 citations, averaging 35.34 citations per paper. The University of Minnesota ranked second, with 48 publications and 4,234 citations, averaging 88.21 citations per paper. The University of Michigan followed closely with 47 publications and 2,738 citations, averaging 58.26 citations per paper. The University of Minnesota had the highest total citation number, totaling 4,234, while the Baylor College of Medicine had the highest average number of citations, totaling 161.82.

#### 3.1.4 Analysis by Author

**Fig. (4):**
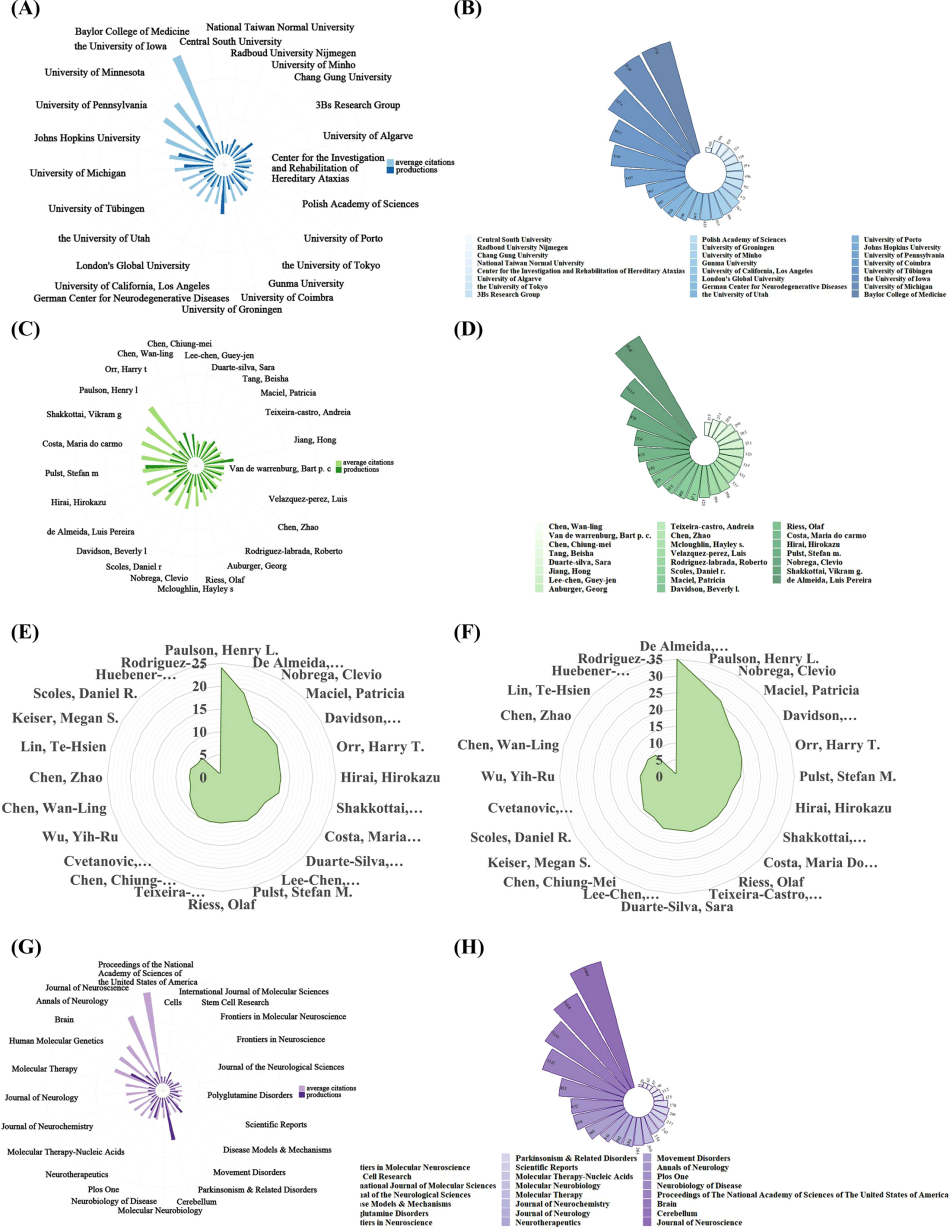
The institutions, authors and journals contributing to the studies related to treatment to polyQ SCA. (**A**) The total number and average citation number of studies related to treatment to polyQ SCA in top 25 most productive institutions. (**B**) The total citation number of studies related to treatment to polyQ SCA in top 25 most productive institutions. (**C**) The total number and average citation number of the top 25 most productive authors in the field of treatment to polyQ SCA. (**D**) The total citation number of the top 25 most productive authors in the field of treatment to polyQ SCA. (**E**) The h-index of the top 25 most productive authors in the field of treatment to polyQ SCA. (**F**) The g-index of the top 25 most productive authors in the field of treatment to polyQ SCA. (**G**) The total number and average citation number of studies related to treatment to polyQ SCA in top 25 most productive journal. (**H**) The total citation number of studies related to treatment to polyQ SCA in top 25 most productive journal.

The total number of authors who participated in studies on the treatment of polyQ SCA was 169. The top 25 contributing authors are shown in Figs. (**4C**, **4D**) and in Table **4**. As depicted in Table **4**, Luis Pereira de Almeida was the most prolific author, with 38 published papers with a total of 1,541 citations, averaging 40.55 citations per paper. Patricia Maciel was the second most prolific author, with 27 studies and a total of 492 citations, averaging 18.22 citations per paper. Clevio Nobrega ranked third, having published 26 studies with a total of 879 citations, averaging 33.81 citations per paper. To evaluate the influence of the publishing authors in this field, the h-index and g-index were evaluated (Figs. **4E**, **4F**). Henry L. Paulson had the highest h-index, followed by Luis Pereira de Almeida and Clevio Nobrega, with the second and third highest h-index, respectively. Regarding the g-index, Luis Pereira de Almeida ranked the highest, followed by Henry L. Paulson and Clevio Nobrega, respectively.

#### 3.1.5 Analysis by Source Journal

The papers related to the treatment of polyQ SCA were published in 38 journals, with the top 25 most productive journals presented in Figs. (**4G**, **4H**) and in Table **5**. Among the journals, *Cerebellum* emerged as the leading journal in terms of the number of publications, publishing 58 studies with a total of 1,418 citations, averaging 24.45 citations per paper. Following closely, *Human Molecular Genetics* ranked second with 38 studies and a total of 2,274 citations, averaging 59.84 citations per paper. *Movement Disorders* was the third most productive journal, with 29 publications and a total of 566 citations, averaging 19.52 citations per paper.

### 3.2 Science Mapping of Publications on polyQ SCA Treatment

#### 3.2.1 Bibliographic Coupling

To evaluate the connections and similarities among publications in this field, bibliographic coupling was performed using VOSviewer. Figs. (**5A**-**5D**) depicts the results of the bibliographic coupling analysis. In the bibliographic coupling network by country, the top five countries with the highest total link strength were the United States (total link strength: 270,629), Portugal (total link strength: 154,381), Germany (total link strength: 98,682), Japan (total link strength: 88,596), and the Netherlands (total link strength: 70,530).

The results of the bibliographic coupling analysis by institutions showed that the top five institutions with the highest total link strength were the University of Coimbra (total link strength: 96,032), the University of Michigan (total link strength: 94,330), the University of Minnesota (total link strength: 61,769), the University of Minho (total link strength: 59,635), and the University of Tübingen (total link strength: 52,338).

In the bibliographic coupling network by author, the top five authors with the highest total link strength were Luis Pereira De Almeida (total link strength: 120,567), Clevio Nobrega (total link strength: 101,188), Henry L Paulson (total link strength: 86,371), Patricia Maciel (total link strength: 81,972), and Sara Duarte-Silva (total link strength: 56,075).

In the bibliographic coupling network by journal, the five most prominent journals with the highest total link strength were *Cerebellum* (total link strength: 18,914), *Human Molecular Genetics* (total link strength: 16,781), *Molecular Neurobiology* (total link strength: 14,938), *Neurobiology of Disease* (total link strength: 14,800), and *Brain* (total link strength: 13,862).

#### 3.2.2 Co-authorship Analysis

**Fig. (6):**
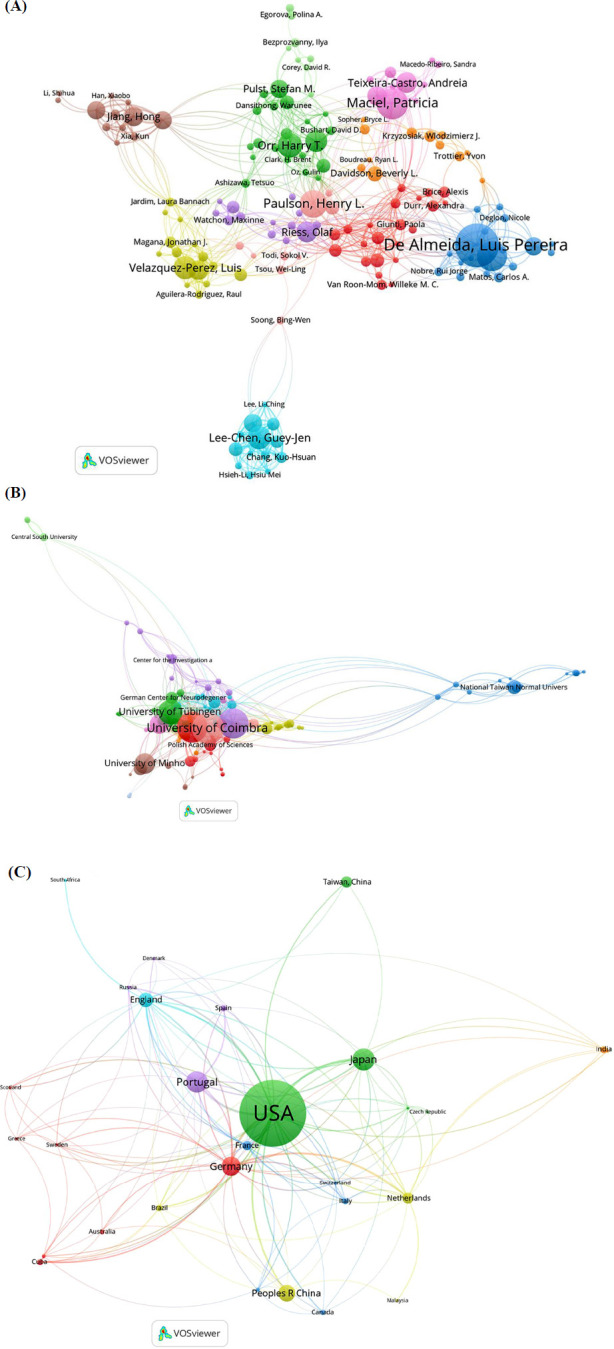
Mapping of co-authorship analysis of studies related to treatment of polyQ SCA (**A**) Mapping of 154-author co-authorship analysis of studies related to treatment of polyQ SCA. (**B**) Mapping of 111-institution co-authorship analysis of studies related to treatment of polyQ SCA. (**C**) Mapping of 29-country co-authorship analysis of studies related to treatment of polyQ SCA.

To evaluate the interconnections among studies based on co-authorship, we conducted a co-author analysis using VOSviewer (Figs. **6A**-**6C**). In the co-authorship analysis, the top five authors with the highest total link strength were Luis Pereira de Almeida (total link strength: 123), Guey-Jen Lee-Chen (total link strength: 101), Chiung-Mei Chen (total link strength: 92), Wan-Ling Chen (total link strength: 83), and Patricia Maciel (total link strength: 81).

In the co-authorship network by institution, the top five institutions with the highest total link strength were the University of Minnesota (total link strength: 82), the University of Tübingen (total link strength: 78), the University of Michigan (total link strength: 72), German Center for Neurodegenerative Diseases (total link strength: 70), and the University of Coimbra (total link strength: 67).

In the co-authorship analysis by country, the top five countries with the highest total link strength were the United States (total link strength: 184), Germany (total link strength: 117), Portugal (total link strength: 89), England (total link strength: 72), and France (total link strength: 62).

#### 3.2.3 Co-citation Analysis

**Fig. (7):**
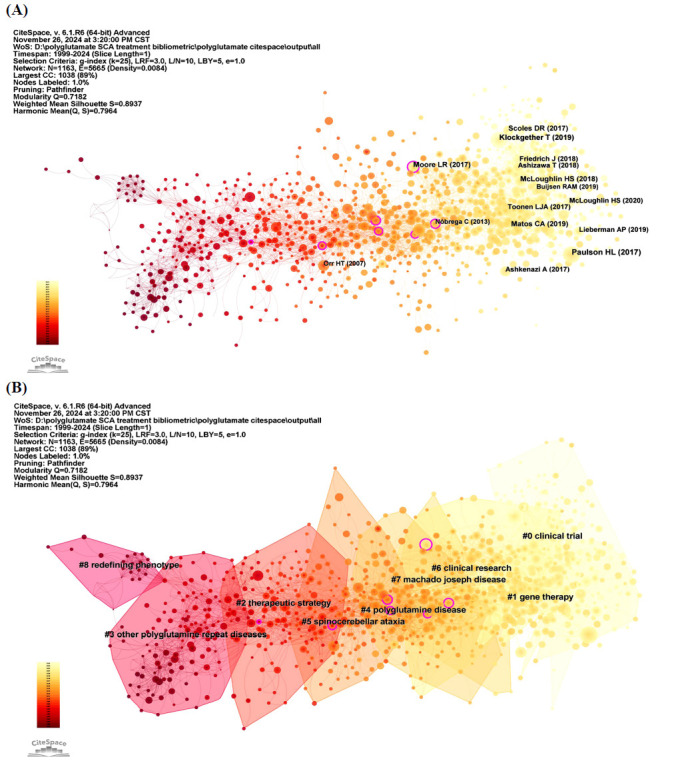
Reference co-citation network mapping of the studies of treatment to polyQ SCA. (**A**) Reference co-citation network of the studies of treatment to polyQ SCA. (**B**) Cluster analysis of reference co-citation network of the studies of treatment to polyQ SCA.

To evaluate the connections among studies based on the co-citation numbers, a co-citation analysis was performed using CiteSpace producing 1,163 nodes and 5,665 edges (Fig. **7A**). The top five references with the highest citation frequency were: “Polyglutamine spinocerebellar ataxias - from genes to potential treatments” [1], “Spinocerebellar ataxia” [58], “Machado-Joseph disease/spinocerebellar ataxia type 3: lessons from disease pathogenesis and clues into therapy” [6], “Evaluation of antisense oligonucleotides targeting ATXN3 in SCA3 mouse models” [59], and “Oligonucleotide therapy mitigates disease in spinocerebellar ataxia type 3 mice” [9]. Similarly, the top five references with the highest centrality were: “Sp1 and TAFII130 transcriptional activity disrupted in early Huntington's disease” [60], “Duplication of Atxn1l suppresses SCA1 neuropathology by decreasing incorporation of polyglutamine-expanded ataxin-1 into native complexes” [61], “Allele-specific silencing of mutant huntingtin and ataxin-3 genes by targeting expanded CAG repeats in mRNAs” [62], “Evaluation of antisense oligonucleotides targeting ATXN3 in SCA3 mouse models” [59], and “HDAC inhibitor sodium butyrate reverses transcriptional downregulation and ameliorates ataxic symptoms in a transgenic mouse model of SCA3” [63]. Additionally, cluster analysis was performed, and the 11 largest clusters are illustrated in Figs. (**7B**, **8A**) as module plots and timeline plots, respectively. The top five largest clusters were “clinical trial”, “gene therapy”, “therapeutic strategy”, “other polyglutamine repeat diseases”, and “polyglutamine disease”.

#### 3.2.4 Reference Burst Analysis

Fig. (**8B**) presents the 25 references that exhibited the strongest citation bursts between 1999 and 2024. The three references with the highest citation burst strengths were “Spinocerebellar ataxia” [58], “Polyglutamine spinocerebellar ataxias: from genes to potential treatments” [1], and “Trinucleotide repeat disorders” [64]. Additionally, the references “Oligonucleotide therapy mitigates disease in spinocerebellar ataxia type 3 mice” [9], “Machado-Joseph disease/spinocere-bellar ataxia type 3: lessons from disease pathogenesis and clues into therapy” [6], “Spinocerebellar ataxia” [58], and “ Antisense oligonucleotide-mediated ataxin-1 reduction prolongs survival in SCA1 mice and reveals disease-associated transcriptome profiles” [7], “Pathogenesis of SCA3 and implications for other polyglutamine diseases” [3], “Polyglutamine repeats in neurodegenerative diseases” [65], “Spinocerebellar ataxias: prospects and challenges for therapy development” [2], “Genetics, mechanisms, and therapeutic progress in polyglutamine spinocerebellar ataxias” [66] continued to attract significant attention until 2024.

### 3.3 Keyword Analysis

#### 3.3.1 Keywords Co-occurrence Analysis

The co-occurrence network analysis of keywords is depicted in Fig. (**9A**). The network, comprised 540 nodes and 4,589 edges. The most frequently occurring keywords included “spinocerebellar ataxia”, “Machado-Joseph disease”, “Huntington’s disease”, “mouse model”, and “CAG repeat”. Additionally, “Machado-Joseph disease”, “dominant cerebellar ataxia”, “cerebellar ataxia”, “Huntington’s disease”, and “polyglutamine disease” was identified as the keywords with the highest centrality.

To illustrate the timeliness of the keywords, both a timeline plot and a module plot were conducted. According to the cluster analysis, there were 11 clusters in total, with the five largest shown in Figs. (**9B**, **10A**), including: cluster #0: polyglutamine diseases, #1: mouse model, #2: polyglutamine spinocerebellar ataxia, #3: Machado-Joseph disease, and #4: spinocerebellar ataxia.

#### 3.3.2 Keywords Burst Analysis

To illustrate the start and end times of significant keywords associated with the treatment of polyQ SCA, a citation burst analysis was performed using CiteSpace software. The 25 keywords with the strongest citation bursts are displayed in Fig. (**10B**). The top five keywords based on burst strength were “neuronal intranuclear inclusion”, “expanded polyglutamine”, “transgenic mice”, “cell death”, and “antisense oligonucleotide”. Furthermore, “repeat”, “dysfunction”, “mutant ataxin 3”, and “identification” were identified as the most recent keywords with sustained interest until 2024.

#### 3.3.3 Alluvial Flow Plot of Keywords

##### 3.3.3.1 Alluvial Flow Plot of Keywords in Different Years

**Fig. (11):**
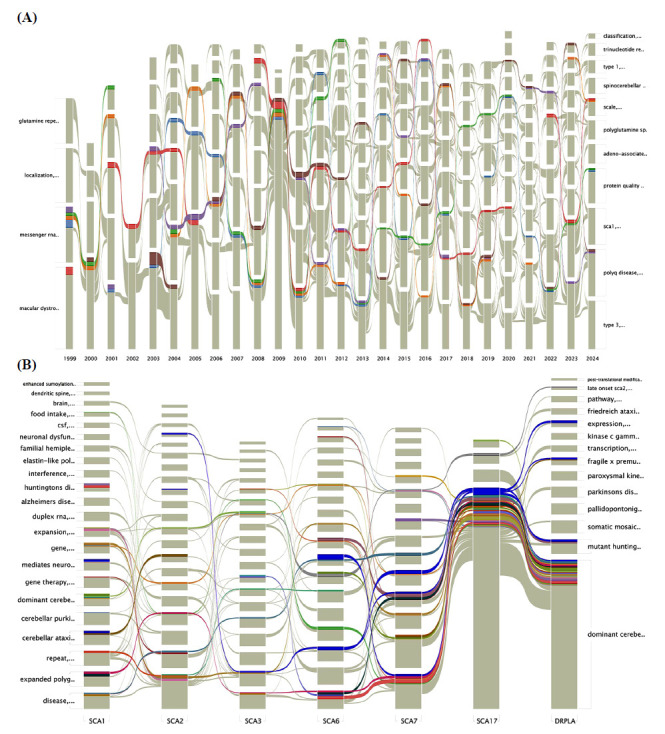
Alluvial plot of keywords in studies of treatment to polyQ SCA. (**A**) The keywords alluvial map of studies of treatment to polyQ SCA from 1999 to 2024. (**B**) The keywords alluvial map of studies of treatment to polyQ SCA in different types of SCA.

To illustrate the timeline of keyowords related to the treatment of polyQ SCA, an alluvial flow map was constructed. The data from the co-occurrence analysis of keywords, conducted using CiteSpace, were input into an alluvial generator (Fig. **11A**). The five keywords with the longest duration of relevance included “Huntington’s disease”, “Machado-Joseph disease”, “CAG repeat”, “cerebellar ataxia”, and “dominant cerebellar ataxia”.

##### 3.3.3.2 Alluvial Flow Plot of Keywords for Different Types of polyQ SCA

To identify the temporal patterns in hotspots across the various types of polyQ SCA, an alluvial flow map was plotted (Fig. **11B**). Thirty keywords that were common to all types of polyQ SCA were highlighted in different colors. These keywords included “Alzheimer’s disease”, “amyotrophic lateral sclerosis”, “androgen receptor”, “ataxia”, “cag repeat”, “cag repeat expansion”, “calcium channel”, “causes cerebellar dysfunction”, “cerebellar ataxia”, “degeneration”, “dentatorubral pallidoluysian atrophy”, “disease”, “dominant cerebellar ataxia”, “double blind”, “expanded polyglutamine”, “expansion, expression”, “gene”, “hereditary ataxia”, “Huntington’s disease”, “Machado Joseph disease”, “model”, “mouse model”, “neurodegeneration”, “neuronal intranuclear inclusion”, “nuclear localization”, “polyglutamine disease”, “polyglutamine expansion”, “spinocerebellar ataxia” and “trinucleotide repeat”.

## 4 DISCUSSION

### 4.1 Trends in PolyQ SCA Treatment

In this study, 1,420 publications were identified from the WOSCC database from its inception to December 2, 2024. The number of publications on the treatment of polyQ SCA steadily increased from 1999 to 2024, suggesting sustained momentum in this research area. Notably, the largest number of publications and citations occurred between 2017 and 2021, likely reflecting the significant achievements published during this period. The work of Paulson *et al*. [1], which summarizes the clinical, pathological, physiological, and molecular aspects of polyQ SCA, laying the foundation for the exploration of new therapeutic approaches, garnered the largest number of citations. The study by Klockgether T [58], which addresses the pathogenic genes, molecular mechanisms, pathology, clinical phenotypes, and therapeutic strategies of SCA, ranked second in terms of the number of citations from 2017 to 2021. Matos CA [6] reported the pathogenic and therapeutic aspects of SCA3, ranking third in terms of the number of citations. The citation count declined slightly after 2021, likely owing to the shorter period available for these publications to accumulate citations. Based on this analysis, we infer that more in-depth studies on polyQ SCA treatment are expected in the coming years.

### 4.2 Distribution of Research on PolyQ SCA Treatment

The distribution of publications across different types of polyQ SCA was also analyzed. Based on the number of publications and citations, SCA3 accounted for the largest proportion of research on polyQ SCA treatment. This may be because SCA3 is the most prevalent form of SCA worldwide [58, 67]. Additionally, although the number of publications for the various subtypes of SCAs varies, publications evaluating SCA3 have increased most rapidly, suggesting that research on SCA3 is a central focus within the field.

This study evaluated the most influential countries, institutions, and authors with respect to research on polyQ SCA treatment based on the number of publications and citations. The United States was the leading contributor, ranking first for both the number of publications and total citations. Additionally, the network analysis revealed that the United States had the highest number of international collaborations and received the most citations from abroad. Portugal and Japan also made significant contributions in terms of publication volume. Although some Asian countries, such as China and Japan, had a large number of publications, their average citation counts were relatively low, suggesting the need for improvement in research quality. In contrast, some European countries, such as England and Germany, despite having fewer publications, achieved high average citation counts, reflecting the quality of their research.

The most productive institutions were primarily located in Europe and the United States. The University of Coimbra ranked first for the number of publications, while Baylor College of Medicine had the highest average citation count among the top 25 most productive institutions. However, few publications from Asian institutions were identified. Network analysis of institutional collaborations revealed that institutions in Europe and the United States have engaged in more extensive collaboration, both in terms of the number of publications and the number of citations, compared to their Asian counterparts. This disparity suggests a significant gap between institutions in developing countries and their European and American counterparts in this academic field. Consequently, future research should focus on improving the quality of publications and promoting stronger collaborations between Asian institutions and institutions in other regions and countries.

To assess the academic productivity and impact of the authors, the h-index and g-index were evaluated. Henry L. Paulson, whose research on polyQ diseases garnered the highest citation count, was identified as the author with the highest h-index and g-index. His work, which systematically explores the etiology, pathogenesis, diagnosis, and treatment of polyQ diseases, has had a significant impact on the field [58]. Luis Pereira de Almeida ranked second for both the h-index and g-index, and he was the most prolific author in terms of publication output. His research primarily focuses on the mechanisms and treatments of polyQ SCA. He demonstrated that autophagy plays a crucial role in the pathology of SCA2 and suggested that it could serve as a potential therapeutic target by modulating molecules in this pathway [68]. Furthermore, his studies on the mutual regulation between ataxin-2 and ataxin-3, the pathogenic proteins of SCA2 and SCA3, may open new avenues for treatment [69]. Recently, Luis Pereira de Almeida proposed that the caffeine's interaction of caffeine with the adenosine A(2A) receptor could alleviate symptoms in mice with SCA3 [70]. Both of these prolific authors have established academic reputations and have made substantial contributions to the advancement of research on polyQ SCA. In general, researchers in Europe and the United States produced more publications with greater citation impact than researchers in Asia. Moreover, collaboration among authors from Europe and the United States has been more pronounced than that of authors from Asian countries.

The sources of these publications also merit attention. *Cerebellum* emerged as the journal with the highest number of publications in this field. Other notable journals included *Human Molecular Genetics* and *Movement Disorders*.

### 4.3 The Focus and Future Direction of Research on PolyQ SCA Treatment

To assess the hotspots and future research directions in polyQ SCA treatment, analysis of references and keywords was performed. The analysis of references identified that the highly cited papers were key contributors to this field. Additionally, the analysis of keywords revealed that terms with high citation counts represent emerging topics in this area. Based on the analysis of keywords and references, the main research hotspots were identified as “new SCA types”, “Huntington’s disease”, “clinical trial”, “gene therapy”, “disease models”, and “aggregation clearance therapy”.

#### 4.3.1 New SCA Subtypes

The co-occurrence analysis of keywords revealed that “spinocerebellar ataxia”, “dominant cerebellar ataxia”, and “cerebellar ataxia” were the most frequently occurring terms in this field, indicating that SCA is a prominent research topic. SCA is a group of heterogeneous disorders, and over 40 genetically distinct subtypes have been identified thus far [58]. Recent discoveries have implicated several genes, including SCA37/DAB1 [71], SCA45/FAT2 [72], SCA46/ PLD3 [73], SCA47/PUM1 [74], SCA48/STUB1 [75], SCA50/ NPTX1 [76], SCA25/PNPT1 [77], SCA49/SAM9DL [78], SCA27B/FGF14 [79], and SCA51/THAP11 [4, 80]. Most of these newly identified subtypes are caused by point mutations, while SCA51/THAP11 results from a CAG repeat expansion in exon 1 of the *THAP11* gene [4]. The THAP11 protein plays a role in regulating the cell cycle, proliferation, transcription, and cobalamin metabolism [81]. As the identification of pathogenic genes for SCA expands, research on new subtypes, such as SCA51/THAP11, may lead to the discovery of novel therapeutic targets for polyQ SCAs.

#### 4.3.2 Huntington’s Disease

The keyword analysis revealed that “Huntington’s disease”, “CAG repeat”, and “CAG repeat expansion” were frequently occurring terms in research on the treatment of polyQ SCA, indicating that research on other polyQ diseases, such as Huntington’s disease, is a prominent topic in this field. Huntington’s disease, a progressive neurodegenerative disorder, is caused by a CAG repeat in the gene encoding huntingtin, leading to an abnormally long polyglutamine repeat in the huntingtin protein [82]. This disorder shares several features with polyQ SCA, including aggregation formation, abnormal protein processing, cellular toxicity, delayed onset, and selective neuronal vulnerability [82]. Huntington’s disease has served as a model for studying other polyQ diseases, particularly polyQ SCA. Therefore, research on Huntington’s disease may provide insights into potential therapeutic strategies for polyQ SCA.

#### 4.3.3 Clinical Trial

The reference co-occurrence analysis identified “clinical trial” as a key cluster of references. In recent years, most research on the pathogenic mechanisms and promising therapeutic strategies has been conducted using cell or animal models, with clinical studies on the treatment of polyQ SCA being relatively limited. However, certain disease-modifying drugs, such as riluzole [21, 22], valproic acid [25], and lithium carbonate [29, 30], have shown encouraging results in clinical trials. Symptomatic treatments, including mesenchymal stem cells [83, 84] and transcranial magnetic stimulation [37, 39], have also yielded promising outcomes in specific types of polyQ SCA. Notably, the most promising therapeutic advancements for SCA involve strategies that reduce or silence disease-causing genes, although these approaches have not yet been extensively studied in clinical trials. It is anticipated that gene-reducing and gene-silencing strategies may transition from laboratory settings to clinical research in the near future.

#### 4.3.4 Gene Therapy

The reference co-occurrence analysis identified “gene therapy” as a key cluster of references, suggesting that gene therapy may be a hot topic in the field of polyQ SCA treatments. For genetic diseases such as polyQ SCA, the most compelling therapeutic targets are the pathogenic genes, regardless of the type of mutation and the pathogenic mechanism. In recent years, many promising gene therapies have been investigated in preclinical studies, showing satisfactory results. RNA interference strategies, such as ASOs, miRNAs, siRNAs, and shRNAs, have shown promising results in cellular and animal models of many polyQ diseases. However, research on gene-editing strategies, such as CRISPR/Cas9 [85], is limited. It is expected that more studies on gene therapies for poly SCA will be conducted and published in the near future.

#### 4.3.5 Disease Models

Keyword analysis revealed that “mouse model” and “model” are hot topics in polyQ SCA research. Animal models are important for studying the molecular and phenotypic aspects of polyQ SCA. There are many animal models for polyQ SCAs. Mouse models are similar in molecular, anatomical, and physiological parts of humans, so they are widely used in research on the treatment of polyQ SCAs [86]. However, it is difficult to model these late-onset diseases in mice because of the short lifespan of these animals. Nevertheless, advances in gene editing methods have partially overcome this hindrance, allowing mutant genes to be overexpressed in mouse models [87]. In recent years, transgenic mouse models of SCA1 [88], SCA2 [89], SCA3 [90], SCA6 [91], SCA7 [92], SCA17 [93] and DRPLA [94] have been generated. More studies utilizing mouse models of polyQ SCA will expand our understanding of the pathogenesis and potential treatment of this group of disorders.

#### 4.3.6 Aggregation Clearance Therapy

From the analysis of the keywords, “neuronal intranuclear inclusion” was the word with the highest burst strength and exhibited the highest burst strength, indicating its significance as a hot topic in polyQ SCA research. For polyQ SCA, the common pathological hallmark is the accumulation of pathogenic protein in the neurons, mostly within the nuclei of neurons [58]. Since pathogenic protein aggregation is central to polyQ SCAs pathology, numerous studies have sought to identify strategies to inhibit these processes, such as enhancing protein quality control pathways and conducting unbiased small-molecule screens [2]. Various molecules have shown success in reducing polyQ protein aggregation, including UL97 kinase [95] and the heat-shock protein DNAJC8 [96]. These findings suggest that reducing neuronal intranuclear inclusions may become a key research focus for future polyQ SCAs treatments.

### 4.4 Strengths and Limits

We utilized various bibliometric tools and methods to perform a comprehensive analysis of studies on polyQ SCA treatment. In order to conduct a more comprehensive analysis, four bibliometric tools were used. HistCite was applied to trace the development history of the field [55]. VOSviewer is suitable for analyzing and visualizing the overall structure of research fields [56]. CiteSpace facilitated the visualization of time series [54]. The R package Bibliometrix, known for its powerful functionality, proved suitable for analyzing multiple fields [57]. The strengths and limitations of these approaches are summarized in Table **6**. Additionally, graphs, networks, charts, maps, and other visual analyses were used to effectively present the findings.

Despite providing a comprehensive bibliometric analysis, this study has certain limitations. First, the literature was exclusively sourced from the Web of Science (WoS) database, which may not encompass all relevant research in this field. Second, the omission of specific keywords by some authors may have resulted in an incomplete representation of research hotspots. Finally, studies published after the search date were not included, potentially introducing an information lag.

## CONCLUSION

This bibliometric study provides an overview of the global status and future directions in research on the treatment of polyQ SCA. The United States emerged as the leading contributor, based on the number of publications. Prominent journals, including *Cerebellum*, *Human Molecular Genetics*, and *Movement Disorders,* accounted for the highest proportion of publications and citations in this field. Furthermore, studies on Machado-Joseph disease, which had the highest production and citation numbers, have garnered substantial attention. It is anticipated that research on the treatment of polyQ SCA will continue to grow in the coming years, with future studies anticipated to focus on newly discovered subtypes of polyQ SCA and other similar neurodegenerative diseases, such as HD, clinical research on gene-reducing or gene-silencing strategies, as well as research on animal models of polyQ SCA and aggregation clearance therapy.

## AUTHORS’ CONTRIBUTIONS

The authors confirm their contribution to the paper as follows: SD, LP, ZC and MD contributed to the research design, data collection and analysis, and manuscript writing. Specifically, SD designed the research and conducted the data collection and analysis. LP assisted with data analysis, while ZC contributed to the data collection. SD and ZC wrote the manuscript in consultation with LP, MD and CT. YG, LH, QW, RQ and HJ assisted with reviewing, editing the references, and revising the draft. All authors contributed to the manuscript and approved the final version.

## Figures and Tables

**Fig. (1):**
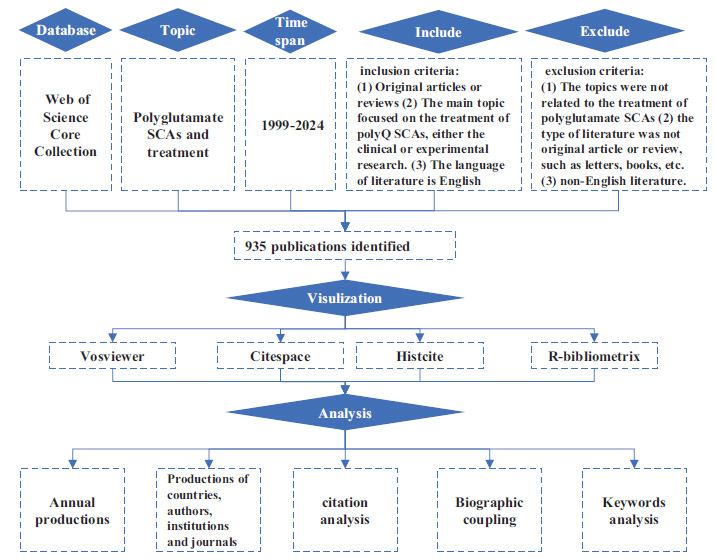
Flowchart depicting the literature search and screening process.

**Fig. (5):**
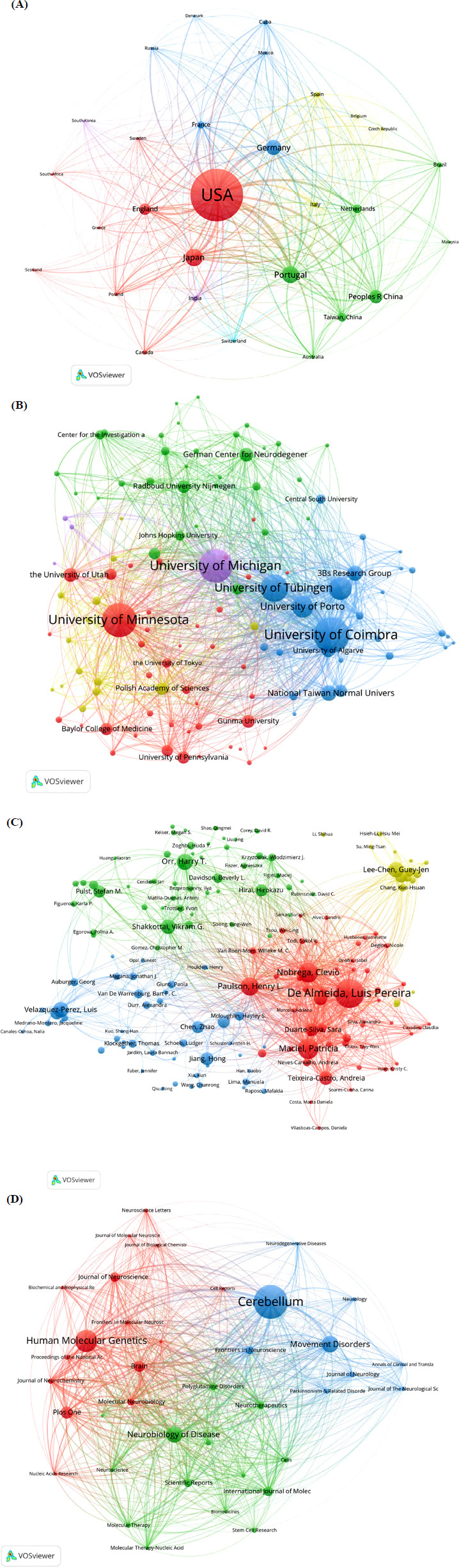
Bibliographic coupling analysis of studies related to treatment of polyQ SCA (**A**) bibliographic coupling network of 29 countries on treatment of polyQ SCA. (**B**) Bibliographic coupling network of 116 institutions on treatment of polyQ SCA. (**C**) Bibliographic coupling network of 169 authors on treatment of polyQ SCA. (**D**) Bibliographic coupling network of 38 journals on treatment of polyQ SCA.

**Fig. (8):**
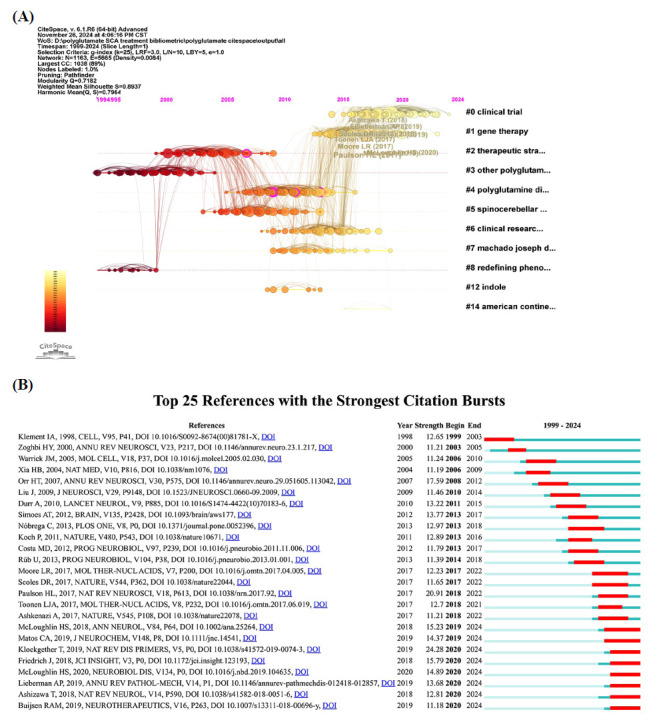
Time-line plot and reference burst analysis of the studies of treatment to polyQ SCA. (**A**) Timeline-view of reference co-citation network of the studies of treatment to polyQ SCA. (**B**) Top 25 references with strongest citation bursts in the field of treatment to polyQ SCA.

**Fig. (9):**
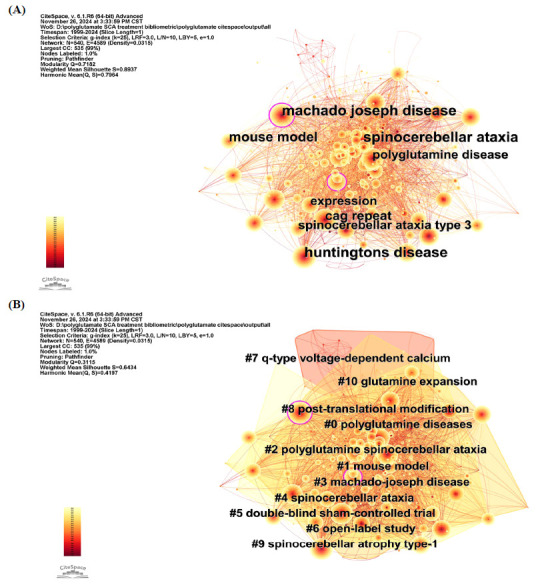
Keywords co-occurrence network of the studies of treatment to polyQ SCA. (**A**) Keywords co-occurrence network of the studies of treatment to polyQ SCA. (**B**) Cluster analysis of keywords co-occurrence network of the studies of treatment to polyQ SCA.

**Fig. (10):**
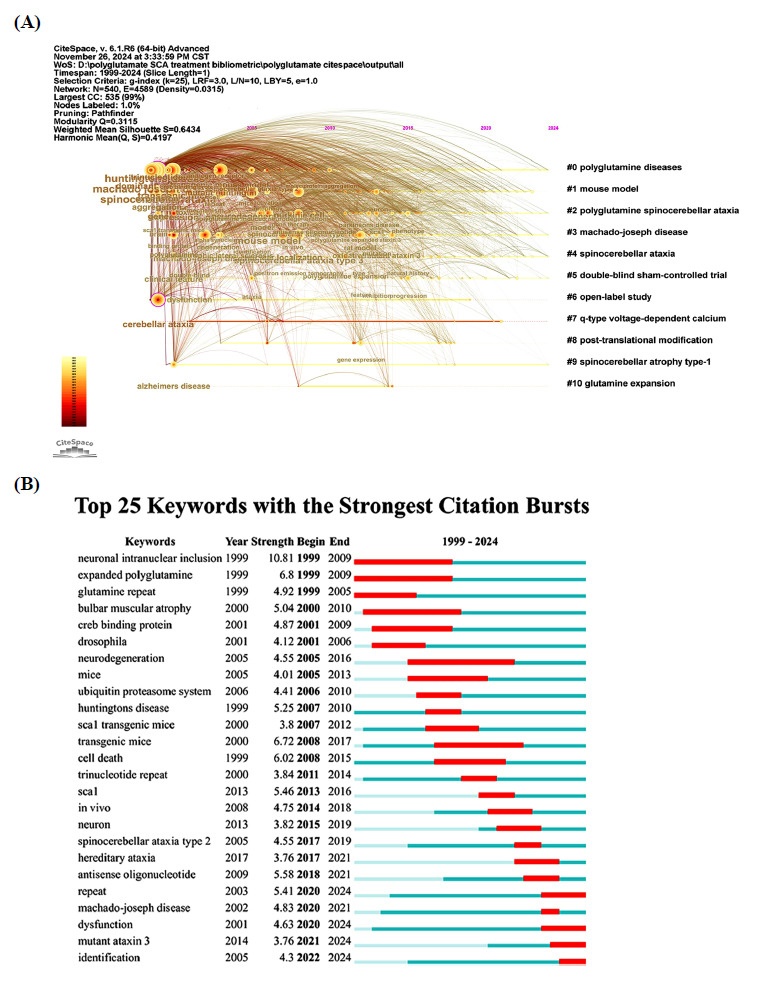
Time-line plot and keywords burst analysis of the studies of treatment to polyQ SCA. (**A**) Timeline-view of keywords co-occurrence network of the studies of treatment to polyQ SCA. (**B**) Top 25 keywords with strongest citation bursts in the field of treatment to polyQ SCA.

**Table 1:** Search terms used for the literature search in Web of Science.

**Topic**	Search Strategy*	**Article Count**	**Article Included**
Treatment of polyQ SCAs	(#9) AND #11	1420	935
Treatment of SCA1	(#1) AND #11	484	191
Treatment of SCA2	(#2) AND #11	206	140
Treatment of SCA3	(#3) AND #11	616	445
Treatment of SCA6	(#4) AND #11	103	67
Treatment of SCA7	(#5) AND #11	90	87
Treatment of SCA17	(#6) AND #11	43	33
Treatment of DRPLA	(#8) AND #11	136	58
Treatment of THAP11	(#9) AND #11	7	0

**Note**: #1: (((((TS=("Spinocerebellar Ataxia Type 1")) OR TS=("Spinocerebellar Ataxia 1")) OR TS=("Spinocerebellar Atrophy I")) OR TS=("Olivopontocerebellar Atrophy I")) OR TS=("Cerebelloparenchymal Disorder I")) OR TS=("SCA1"). #2: (((((TS=(“Spinocerebellar Ataxia Type 2”)) OR TS=(“Olivopontocerebellar Atrophy 2”)) OR TS=(“Spinocerebellar Atrophy 2”)) OR TS=(“Wadia Swami Syndrome”)) OR TS=(“Spinocerebellar Ataxia 2”)) OR TS=(“SCA2”). #3:(((((((TS=(“Spinocerebellar Ataxia Type 3”)) OR TS=(“Machado Joseph Disease”)) OR TS=(“Machado Joseph Azorean Disease”)) OR TS=(“Azorean Disease”)) OR TS=(“Azorean Neurologic Disease”)) OR TS=(“Nigrospinodentatal Degeneration”)) OR TS=(“Spinocerebellar Ataxia 3”)) OR TS=(“SCA3”). #4: ((TS=(“Spinocerebellar Ataxia Type 6”)) OR TS=(“Spinocerebellar Ataxia 6”)) OR TS=(“SCA6”). #5: (((((TS=(“Spinocerebellar Ataxia Type 7”)) OR TS=(“Spinocerebellar Ataxia 7”)) OR TS=(“OPCA with Macular Degeneration and External Ophthalmoplegia”)) OR TS=(“OPCA with Retinal Degeneration”)) OR TS=(“Olivopontocerebellar Atrophy III”)) OR TS=(“SCA7”). #6: (((TS=(“Spinocerebellar Ataxia 17”)) OR TS=(“Huntington Disease-Like 4”)) OR TS=(“HDL4”)) OR TS=(“SCA17”). #7: (((TS=(“Polyglutamine spinocerebellar ataxia*”)) OR TS=(“polyQ SCA*”)) OR TS=(“Polyglutamine SCA*”)) OR TS=(“polyQ spinocerebellar ataxia *”). #8: ((((TS=(“DRPLA”)) OR TS=(“Dentatorubral Pallidoluysian Atroph*”)) OR TS=(“Naito Oyanagi Disease”)) OR TS=(“Haw River Syndrome”)) OR TS=(“ATN1”). #9: TS=(THAP11). #10: (((((((#8) OR #7) OR #6) OR #5) OR #4) OR #3) OR #2) OR #1. #11: (((TS=(treatment*)) OR TS=(management*)) OR TS=(therap*)) OR TS=(drug*).

**Table 2:** The top 10 most productive countries related to treatment to polyQ SCAs from 1999 to 2024.

**Country**	**Productions**	**Citations**	**Average Citations**
USA	313	17496	55.8978
China	133	2397	18.0226
Japan	105	3773	35.9333
Portugal	102	2923	28.6569
Germany	90	3905	43.3889
England	69	4311	62.4783
Netherlands	52	1548	29.7692
France	45	2287	50.8222
India	33	412	12.4848
Brazil	31	521	16.8065

**Table 3:** The top 25 most productive institutions related to treatment to polyQ SCAs from 1999 to 2024.

**Institutions**	**Productions**	**Citations**	**Average Citations**
University of Coimbra	50	1767	35.34
University of Minnesota	48	4234	88.2083
University of Michigan	47	2738	58.2553
University of Tübingen	38	1927	50.7105
University of Minho	32	599	18.7188
University of Porto	30	862	28.7333
National Taiwan Normal University	22	326	14.8182
3Bs Research Group	21	422	20.0952
German Center for Neurodegenerative Diseases	19	749	39.4211
the University of Utah	19	828	43.5789
Radboud University Nijmegen	18	306	17
University of Algarve	18	396	22
Polish Academy of Sciences	18	472	26.2222
Gunma University	18	633	35.1667
Chang Gung University	17	320	18.8235
University of Pennsylvania	17	1437	84.5294
Baylor College of Medicine	17	2751	161.8235
University of California, Los Angeles	16	633	39.5625
London's Global University	16	645	40.3125
the University of Iowa	16	2274	142.125
University of Groningen	15	567	37.8
Johns Hopkins University	14	963	68.7857
Central South University	13	102	7.8462
Center for the Investigation and Rehabilitation of Hereditary Ataxias	13	328	25.2308
The University of Tokyo	13	406	31.2308

**Table 4:** The top 25 most productive authors in the field of treatment to polyQ SCAs from 1999 to 2024.

**Author**	**Productions**	**Citations**	**Average Citations**
De Almeida, Luis Pereira	38	1541	40.5526
Maciel, Patricia	27	492	18.2222
Nobrega, Clevio	26	879	33.8077
Paulson, Henry l	24	2247	93.625
Orr, Harry t	21	2110	100.4762
Lee-chen, Guey-jen	20	323	16.15
Velazquez-perez, Luis	20	396	19.8
Shakkottai, Vikram g	19	1125	59.2105
Riess, Olaf	18	579	32.1667
Teixeira-castro, Andreia	18	331	18.3889
Duarte-silva, Sara	17	302	17.7647
Jiang, Hong	17	321	18.8824
Rodriguez-labrada, Roberto	17	421	24.7647
Chen, Chiung-mei	16	256	16
Chen, Zhao	16	337	21.0625
Hirai, Hirokazu	16	673	42.0625
Pulst, Stefan m	16	726	45.375
Tang, Beisha	16	290	18.125
Davidson, Beverly l	13	515	39.6154
Chen, Wan-ling	12	175	14.5833
Costa, Maria do carmo	12	581	48.4167
Mcloughlin, Hayley s	12	390	32.5
Scoles, Daniel r	12	471	39.25
Van de warrenburg, Bart p. c	12	231	19.25
Auburger, Georg	11	324	29.4545

**Table 5:** The top 25 most productive journals regarding treatment to polyQ SCAs from 1999 to 2024.

**Journal**	**Productions**	**Citations**	**Average Citations**
Cells	9	26	2.8889
International Journal of Molecular Sciences	17	95	5.5882
Stem Cell Research	9	67	7.4444
Frontiers in Molecular Neuroscience	7	63	9
Frontiers in Neuroscience	14	206	14.7143
Journal of the Neurological Sciences	7	112	16
Polyglutamine Disorders	11	178	16.1818
Scientific Reports	14	243	17.3571
Disease Models & Mechanisms	7	125	17.8571
Movement Disorders	29	566	19.5172
Parkinsonism & Related Disorders	9	217	24.1111
Cerebellum	58	1418	24.4483
Molecular Neurobiology	13	350	26.9231
Neurobiology of Disease	28	832	29.7143
Plos One	21	672	32
Neurotherapeutics	13	451	34.6923
Molecular Therapy-Nucleic Acids	7	254	36.2857
Journal of Neurochemistry	10	384	38.4
Journal of Neurology	10	392	39.2
Molecular Therapy	9	364	40.4444
Human Molecular Genetics	38	2274	59.8421
Brain	20	1243	62.15
Annals of Neurology	10	658	65.8
Journal of Neuroscience	17	1680	98.8235
Proceedings of The National Academy of Sciences of The United States of America	9	1112	123.5556

**Table 6:** Strengths and limits of bibliometric tools.

**Tools/Methods**	**Strengths**	**Limits**
HistCite	Simple operation, Capable of analyzing large-scale datasets.	Only English-language papers could be analysis, can only analyze papers from the Web of Science (WoS) database.
VOSviewer	Effective visual representation, suitable for analyzing and visualizing the overall structure of research fields, relatively simple operation.	Low efficiency in processing large-scale datasets.
CiteSpace	Effective visual representation, capable of processing large-scale datasets, emphasizes the visualization of time series.	Relatively complex operation.
R package Bibliometrix	Powerful functionality and suitable for multiple fields, presents analysis results intuitively.	Dependent on R language environment, and some functions may require integration with other R packages.

## Data Availability

The data for this study were sourced from the Web of Science core collection, with express permission granted.

## LIST OF ABBREVIATIONS

ASOs: Antisense Oligonucleotides
DRPLA: Dentatorubral Pallidoluysian Atrophy
SCA: Spinocerebellar Ataxia
siRNAs: Small Interfering RNAs
